# Editorial Comment to Advanced adrenocortical carcinoma successfully treated with gemcitabine plus capecitabine as second‐line chemotherapy

**DOI:** 10.1002/iju5.12222

**Published:** 2020-09-15

**Authors:** Shuya Kandori

**Affiliations:** ^1^ Department of Urology Faculty of Medicine University of Tsukuba Tsukuba Ibaraki Japan

Yamamoto *et al*. presented the case of a patient with advanced adrenocortical carcinoma (ACC) who was treated successfully with gemcitabine, capecitabine, and mitotane (GC‐M) therapy after etoposide, doxorubicin, cisplatin, and mitotane (EDP‐M) therapy.^1^

ACC is a rare and aggressive malignant neoplasm that typically has a poor prognosis. In cases with metastatic disease, 5‐year survival rates have been reported to range from 0% to 28%.^2^ The First International Randomized Trial in Locally Advanced and Metastatic Adrenocortical Carcinoma Treatment demonstrated improved progression‐free survival (PFS) in patients treated with EDP‐M therapy compared to patients treated with streptozotocin plus mitotane.^3^ EDP‐M therapy was generally accepted as the current first‐line treatment for advanced ACC on the basis of the results of this trial.^1^ Second‐line treatment for advanced ACC, however, is not well established. Based on preclinical data, several targeted therapies for advanced ACC have been investigated.^4^ However, none has yet been shown to definitively improve PFS or overall survival in large randomized trials.

The case report by Yamamoto *et al*. presents a presented with advanced ACC who was treated with GC‐M therapy as a second‐line treatment and achieved long‐term disease control.^1^ In an earlier study, Henning *et al*. reported the efficacy and safety of gemcitabine‐based chemotherapy for patients with advanced ACC.^5^ Henning *et al*. found an objective response rate of 4.9% and a median PFS of 12 weeks (range 1–94 weeks), but a few patients with long‐term disease control were described.^5^ GC‐M therapy would be a therapeutic option for patients who progress under EDP‐M therapy and cannot be enrolled in clinical trials.

## Conflict of interest

The author declares no conflict of interest.
